# Diffuse Alveolar Hemorrhage: A Rare Complication of Severe Hypertension

**DOI:** 10.7759/cureus.33933

**Published:** 2023-01-18

**Authors:** Mohamad Talal Basrak, Muhammad Firas Alhammad, Shahzad Anjum, Mohammad Altermanini, Yasir E Ahmed

**Affiliations:** 1 Emergency Medicine, Hamad Medical Corporation, Doha, QAT; 2 Anesthesia, Hamad Medical Corporation, Doha, QAT; 3 Internal Medicine, Hamad Medical Corporation, Doha, QAT

**Keywords:** uncontrolled hypertension, sudden-onset, sob - shortness of breath, hemoptysis, severe hypertension, diffuse alveolar hemorrhage

## Abstract

Severe hypertension is a rare cause of diffuse alveolar hemorrhage. We reported a case of a 43-year-old woman who presented with shortness of breath, hemoptysis, and severe hypertension. The patient was diagnosed with diffuse alveolar hemorrhage due to severe hypertension which improved after controlling her blood pressure.

## Introduction

Diffuse alveolar hemorrhage (DAH) is a life-threatening condition that must be recognized and treated early to decrease morbidity and mortality. It is known that severe hypertension can cause end-organ damage; however, hemoptysis and DAH could be one rare complication of severe hypertension as described in our case. Controlling blood pressure in this type of patients is the mainstay of treatment.

## Case presentation

A 43-year-old female presented to the emergency department for a first episode of sudden onset shortness of breath and several episodes of hemoptysis. The patient did not have any chest pain, cough, fever, weight loss, hematemesis, hematochezia, or hematuria. The patient did not have any history of exposure to toxic substances, smoking, or alcohol.

Her past medical history was remarkable for hypertension diagnosed five years back. The patient was on medical treatment (Valsartan/Amlodipine 160mg/10mg tablet), but she had discontinued her medication for the last two months. In addition, she had a history of uncontrolled diabetes mellitus type 2.

On physical examination, the patient was tachypneic (respiratory rate 30 per minute) with low oxygen saturation SPO_2_ 90% on room air that improved with oxygen supplementation by 3 L/min nasal cannula to 99%, hypertensive (blood pressure=224/143, tachycardiac (heart rate 105 per minute) and normal temperature. On auscultation, chest is clear, no wheezes or crackles, and normal s1 and s2 without any added sounds or murmurs.

**Figure 1:**
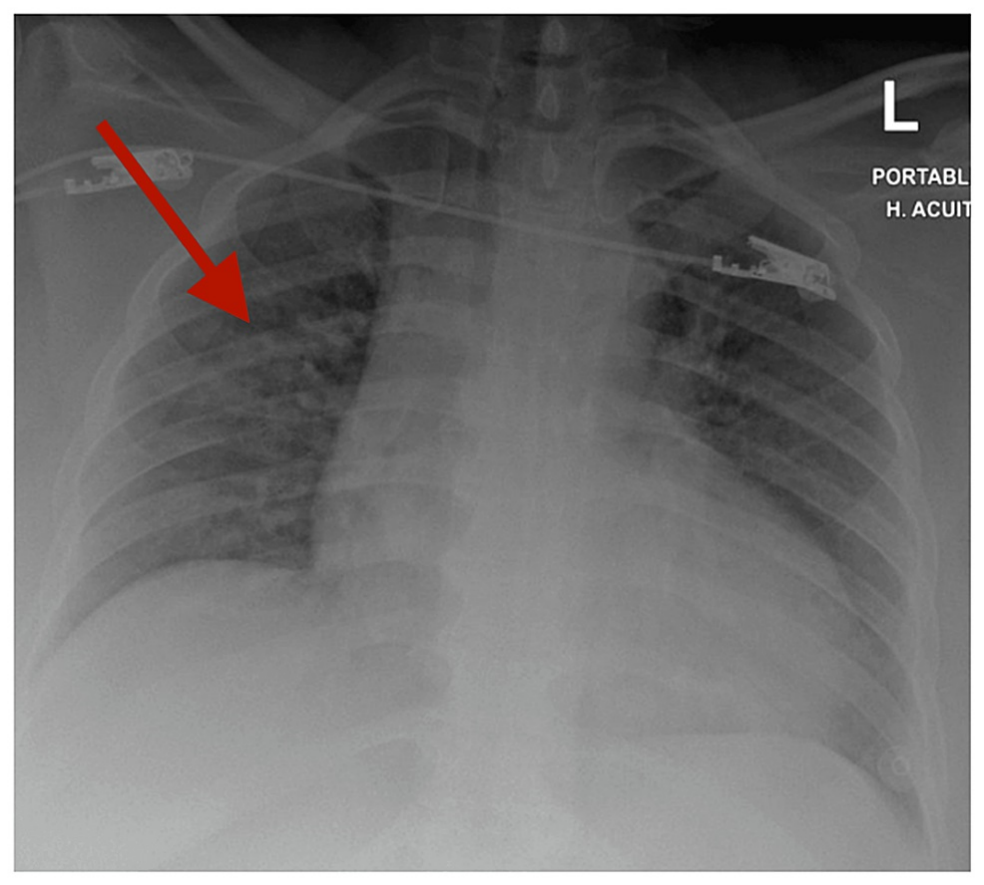
Chest x-ray (prominent broncho vascular markings with focal infiltrates in the right middle zone)

Laboratory blood tests were all within the normal range. Notably, her hemoglobin dropped from 14.7 to 13.6 g/dL and her hematocrit dropped from 43.1% to 40.6%. White blood cells WBC= 8x10^3^/microliter. Procalcitonin=0.05 ng/mL. Normal coagulation studies (INR=0.9, Aptt=26.4 seconds). Renal function tests were within the normal range (urea 5.8 mmol/L, creatinine 83 μmol/L), Na 138 mmol/L, K=4 mmol/L. Chest x-ray (Figure 1) showed prominent broncho-vascular markings with focal infiltrates in the right middle zone and cardiomegaly.

**Figure 2:**
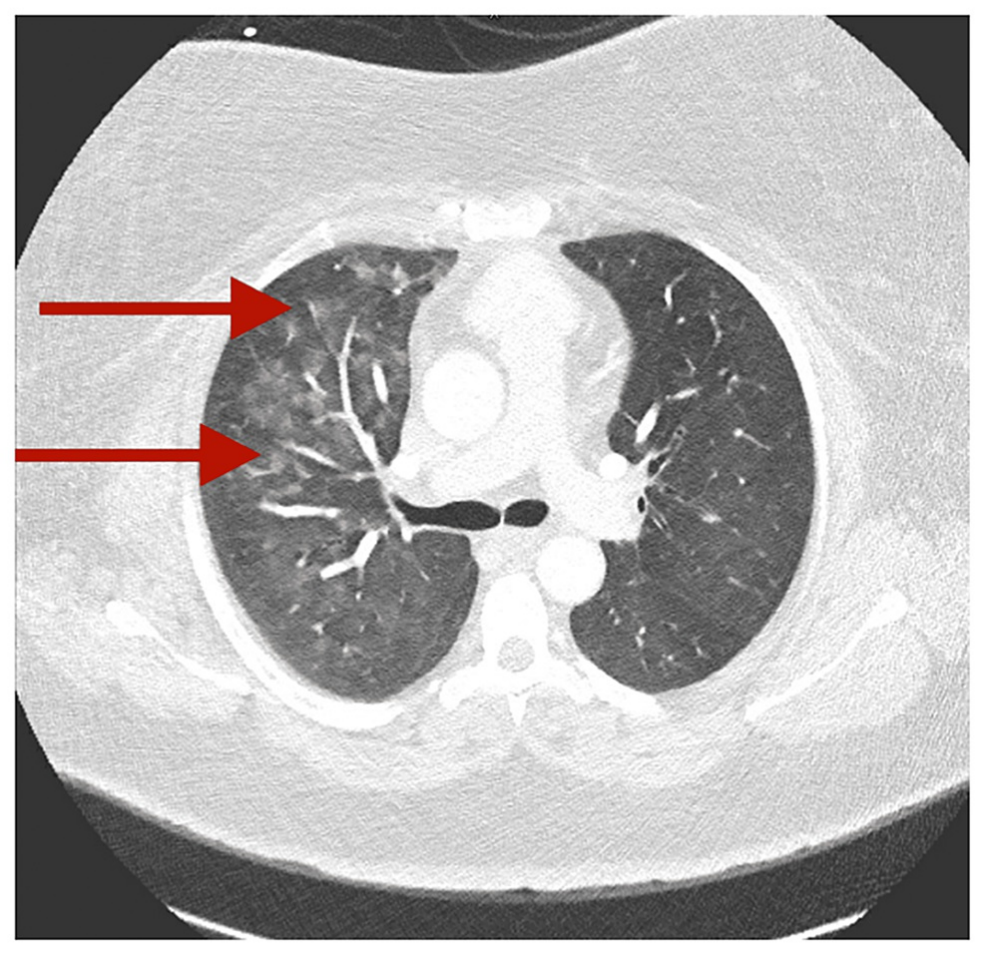
CT chest with contrast (diffuse fluffy ground glass opacities bilaterally involving the right upper and middle lobes)

CT chest with contrast (Figure 2) (pulmonary angiography protocol) showed diffuse fluffy ground glass opacities bilaterally involving the right upper and middle lobes, which suggest alveolar hemorrhage or edema with no evidence of pulmonary embolism.

Echocardiography showed mildly reduced left ventricular systolic function with a calculated ejection fraction of 47%, grade 1 diastolic dysfunction, and normal left atrial pressure. There was mild concentric left ventricular hypertrophy. COVID-19 PCR, acid-fast bacilli smear, tuberculosis PCR and immunology workup were all negative.

During bronchoscopy, mucosa was looking normal without active bleeding; however, sequential lavage taken from the right upper lobe was hemorrhagic, worsening and not clearing. Bronchial wash and bronchial alveolar lavage consisted of reddish and turbid fluid. Microscopy was negative for malignancy and granuloma. Bronchoscopy showed 26% lymphocytes with positive hemosiderin-laden macrophages.

Within a few hours, blood pressure was controlled with three boluses of intravenous labetalol 20mg and Valsartan/Amlodipine 160mg/10mg tablet. Hemoptysis stopped. Shortness of breath improved, and oxygen requirements gradually decreased (blood oxygen saturation 98% on room air). No other drugs were needed. The patient was discharged home after 48 hours on Valsartan/Amlodipine 160mg/10mg tablet. Based on the clinical presentation, imaging, bronchoscopy findings and laboratory blood tests, the patient was diagnosed with DAH as a complication of severe hypertension.

## Discussion

The main pathophysiology involved in DAH is an active local inflammatory process. DAH has a variety of causes most commonly systemic vasculitis, rheumatoid disease, connective tissue disease, drugs, and toxins; however, severe hypertension is extremely a rare cause. Severe hypertension has been reported in the literature as a cause of DAH in only seven case reports (Table 1).

**Table 1:** Previously published similar case reports for patients presenting with hemoptysis and severe hypertension [6].

Case report	Age	Gender	Comorbidities	Blood pressure on admission	Treatment
Hida K et al [1].	34	Male	HTN newly diagnosed	220/135	Antihypertensive medication, Hemodialysis, Prednisolone, Cyclophosphamide
Sato Y et al [2].	26	Male	HTN not on treatment	210/150	Antihypertensive medication
Aithal S et al [3].	38	Male	Smoker	220/120	Antihypertensive medication
Nanba K et al [4].	32	Male	HTN not on treatment	290/150	Antihypertensive medication
Park HS et al [5].	27	Male	None	180/100	Steroids, Antihypertensive medication
Suzuki et al [6].	27	Male	None	200/128	Hemodialysis Plasma exchange, Steroids, Carperitide, Fursemide, Antihypertensive medication
Daniel Ramos et al [7].	51	Male	Smoker	220/130	Antihypertensive medication, Hemodialysis

In this case, the patient was known to have hypertension. She presented to the emergency department with hemoptysis as the main complaint. Detailed history and physical examination led to many investigations to define a certain diagnosis. Laboratory tests excluded any infection or autoimmune disease. The final diagnosis of DAH as a complication of severe hypertension was concluded based on clinical presentation, imaging and bronchoscopy findings. Clinically the patient improved dramatically after controlling her blood pressure within a few hours.

Diagnosis of a DAH depends on history, physical examination, diagnostic imaging, laboratory findings and invasive diagnostic procedures like bronchoscopy. The presence of ≥20% hemosiderin-laden macrophages in bronchioalveolar lavage (BAL) fluid is commonly diagnostic for DAH and closely associated with the severity [8].

How exactly severe hypertension can cause DAH is not fully understood; however, left ventricle dysfunction might have a role in causing an increase in pulmonary vasculature which might lead to pulmonary edema and alveolar hemorrhage [2,3]. Renin, aldosterone, vasopressin, catecholamines and endothelin might be involved in the alveolar-capillary injury. Finally, the bronchopulmonary arteries might have suffered directly from severe systemic hypertension in some patients as the bronchopulmonary arteries derive their blood supply from the systemic and pulmonary circulations or alveolar septal vessels [6].

## Conclusions

DAH should be considered in patients presenting with a sudden onset of shortness of breath, hemoptysis and severe hypertension. Early recognition and efficient management of high blood pressure might be beneficial for clinical improvement and decrease morbidity and hospital stay.
